# Taxonomic and Functional Metagenomic Signature of Turfs in the Abrolhos Reef System (Brazil)

**DOI:** 10.1371/journal.pone.0161168

**Published:** 2016-08-22

**Authors:** Juline M. Walter, Diogo A. Tschoeke, Pedro M. Meirelles, Louisi de Oliveira, Luciana Leomil, Márcio Tenório, Rogério Valle, Paulo S. Salomon, Cristiane C. Thompson, Fabiano L. Thompson

**Affiliations:** 1 Laboratory of Microbiology, Institute of Biology, Federal University of Rio de Janeiro (UFRJ), Rio de Janeiro, Brazil; 2 Center of Technology—CT2, SAGE-COPPE, Federal University of Rio de Janeiro (UFRJ), Rio de Janeiro, Brazil; 3 Institute of Biology, Federal University of Rio de Janeiro (UFRJ), Rio de Janeiro, Brazil; 4 COPPE-Production Engineering Program, Federal University of Rio de Janeiro (UFRJ), Rio de Janeiro, Brazil; Pennsylvania State University, UNITED STATES

## Abstract

Turfs are widespread assemblages (consisting of microbes and algae) that inhabit reef systems. They are the most abundant benthic component in the Abrolhos reef system (Brazil), representing greater than half the coverage of the entire benthic community. Their presence is associated with a reduction in three-dimensional coral reef complexity and decreases the habitats available for reef biodiversity. Despite their importance, the taxonomic and functional diversity of turfs remain unclear. We performed a metagenomics and pigments profile characterization of turfs from the Abrolhos reefs. Turf microbiome primarily encompassed Proteobacteria (mean 40.57% ± s.d. 10.36, N = 1.548,192), Cyanobacteria (mean 35.04% ± s.d. 15.5, N = 1.337,196), and Bacteroidetes (mean 11.12% ± s.d. 4.25, N = 424,185). Oxygenic and anoxygenic phototrophs, chemolithotrophs, and aerobic anoxygenic phototrophic (AANP) bacteria showed a conserved functional trait of the turf microbiomes. Genes associated with oxygenic photosynthesis, AANP, sulfur cycle (S oxidation, and DMSP consumption), and nitrogen metabolism (N_2_ fixation, ammonia assimilation, dissimilatory nitrate and nitrite ammonification) were found in the turf microbiomes. Principal component analyses of the most abundant taxa and functions showed that turf microbiomes differ from the other major Abrolhos benthic microbiomes (i.e., corals and rhodoliths) and seawater. Taken together, these features suggest that turfs have a homogeneous functional core across the Abrolhos Bank, which holds diverse microbial guilds when comparing with other benthic organisms.

## Introduction

Turfs represent one of the most abundant benthic functional groups in the Atlantic, Caribbean, and Pacific reefs; however, there have been a limited number of studies characterizing the microbial composition of turfs [1–7]. Previous studies suggested that microbiomes play a central role in the functions of turfs. However, the majority of these studies were restricted to the Pacific region, and the composition of turf microbiomes remains unknown. As a functional group, turfs are characterized by filamentous and short, upright tufts rich in detritus that form a canopy less than 1 cm high over the substratum [8], [9]. In the Abrolhos Bank, turf is the most abundant group of benthic organisms on the reefs, having an average coverage of 56% (ca. ± s.d. 4.0) over the 8,844 Km^2^ reef area [10]. A marked turf spread has been observed over the last decade, potentially reflecting a phase shift process [11]. In this process, a decrease in the abundance of herbivorous fish leads to an increase in turf cover [12], [13]. Additionally, the spread of turf can be promoted by an increase in temperature and nutrients as a result of local and global environmental changes [14]. The presence of potentially toxic photosynthetic and pathogenic microbes may also favor the spread of turfs in coral reefs, along with a potential increase in photosynthesis and heightened adaptation to changing environmental conditions [15]. For example, bacteria that are pathogenic to corals in the turf can kill the corals, which then serve as substrates for turf spreading [16]. In addition, turf overgrowth above corals can also lead to oxygen depletion, anoxia, and tissue death in corals [17], [18]. Turfs release higher concentration of dissolved organic carbon (DOC) [18–20], which stimulate rapid growth of heterotrophic microbes [19], [21] and may promote shifts towards copiotrophic and potentially pathogenic microbial communities [20]. However, previous studies have not addressed the functional metagenomic diversity of turfs. It is currently unclear whether turfs have a specific stable microbiome or several types of turf microbiomes co-existing in the same reef system.

Turf has been recently defined as a holobiont, or an assemblage of macrorganisms (mainly algae) and microrganisms, representing an unified functional entity [7]. Analogous to the coral holobiont concept, both host and microbiome produce extracellular products that permit the holobiont to closely interact [22]. In the coral holobiont, the host is a Cnidarian. Symbiotic microbes and *Symbiodinium* grow on the coral mucus and inside the coral cells. In contrast, a single host is not evident in turf [23]. The host could be a filamentous cyanobacterium due to their abundance, dimension (large macroscopic filaments), and copious production of exudates (dissolved organic matter) [5]-[7]. However, recent critiques of the holobiont concept have led to substantial turmoil [24] amongst researchers, suggesting that further evidence is required to prove that turf is a holobiont. For instance, the stability of turf assemblages across both different reef locations and seasons is unclear.

Previous researchers have suggested that cyanobacteria are the dominant microbial group in turf, representing more than half of the microbes in turf [25–27]. Turfs in the Mascarene Archipelago (Indian Ocean) are dominated by species of the genera *Hydrocoleum*, *Anabaena*, *Symploca*, *Leptolyngbya*, and *Lyngbya* [27]. Furthermore, cyanobacteria can play a crucial role in the physical structuring of turf due to their filamentous (turf-like) nature. Cyanobacteria significantly contribute to photosynthesis and N cycling in benthic communities.

In contrast with previous studies, Barott and co-workers [1] used 16S rRNA pyrosequencing to estimate that 7,700 different types of bacteria are associated with turf. Approximately 50% of the identified sequences were related to Proteobacteria. Turf also appeared to be a source of phototrophic bacteria (e.g., Rhodobacteraceae), acid-tolerant bacteria (e.g., *Acidovorax*, *Lactobacillus*), and potentially pathogenic bacteria (e.g., *Vibrio* and Bacteroidetes). A recent analysis of 38 turf samples from the Line Islands resulted in an estimated 18,065 Operational Taxonomic Units (OTUs) deemed to be stable symbionts via 16S rRNA pyrosequencing [7]. The most abundant and stable turf symbionts are Alphaproteobacteria of the orders Rhodobacterales, Rhizobiales, and Rhodospirales [7]. Hester et al. introduced the stable and sporadic community concept, wherein bacterial communities are neither ubiquitous nor specific across turf samples [7], [28].

These previous studies inspired us to test the hypothesis that turfs have a conserved genomic and metabolic signature, with similar communities and functional gene repertoires spread across different Abrolhos reef locations and seasons. The assemblage of a diverse metagenome could explain why turfs are becoming a dominant component of reefs. In contrast with previous studies, we applied metagenomics to uncover the major types of metabolism found in turfs. Metagenomics has been used to investigate the microbial metabolic potential across a variety of marine and terrestrial environments [12], [29–31], whereas the majority of turf studies have relied on the diversity analysis of partial 16S rRNA sequences, which cannot realize the functional potential or diverse functional gene repertoire of turf (e.g., photosynthesis, nitrogen metabolism, and sulfur metabolism).

In the present study we tested the following hypotheses for the microbial composition of the turf from the Abrolhos reefs: H1) the taxonomic and H2) functional composition of the turf are conserved in space (three different locations, inside and outside of protected areas) and time (at two different seasons of the year, summer and winter); H3) the abundance of key genes (e.g. oxygenic and anoxygenic photosynthesis, and chemosynthesis) are different among turf and other benthic holobionts (corals and rhodoliths) and seawater. We also performed morphologic and pigment characterization of turf which enabled us to assess their healthy status. The functional diversity assessment obtained by metagenomics allows us to infer potential adaptive advantages that occur during the competition for space amongst the benthic organisms in reef systems.

The conserved nature of the functional composition of turf metagenomes observed in this study hints to a stable core of complementary functions in the turf.

## Materials and Methods

### Study site and sample collection

This study was conducted at the Abrolhos Bank (16°40′,19°40′S/39°10′, 37°20′W) at near-shore and off-shore locations. In total, 19 turf samples were collected by SCUBA divers from 10–15 m depths; eleven samples were collected on March 10, 2013 (austral summer) and eight samples were collected on October 15, 2013 (austral winter). The samples were collected from three reefs sites: (A) Pedra de Leste (PL, 39°2’00”W/17°46’00”S), (B) Parcel dos Abrolhos (PAB, 17°57′32.7″S/38°30′20.3″W), and (C) Archipelago/Mato Verde (AR, 17°96’43”S/38°70’06”W for March, and 17°57′76.4″S/38°41′73.7″W for October) (S1 Fig). The sampling design allowed for spatial (inner vs. outer shelf) and temporal comparisons. We selected locations inside and outside protected marine areas with different degrees of degradation to account for wide environmental variation. The areas located within Abrolhos National Park had higher coral coverage and higher fish biomass than those located outside the park [14]. To characterize the metagenomes and pigment profiles of the nineteen turf samples, we aimed to shed light on the features that enable this multispecies assembly system to be the most abundant benthic organisms in the Abrolhos Bank. Each turf sample was collected a minimum of 10 meters away from the previous. At each site, 2.3 cm^2^ samples of turf were carefully collected from the barren bottom using metal spatulas, without coral tissue scraping. The samples were stored in sterile polypropylene tubes and preserved in liquid nitrogen for further metagenomic and pigment analyses. In addition, turf samples collected in parallel were fixed with glutaraldehyde (1% final conc.) in seawater and further examined with light microscopy. The remaining samples were fixed in 4% formaldehyde to determine their ash-free dry weights. The sampling was authorized by the Brazilian Environmental Agency, Instituto Chico Mendes de Conservação da Biodiversidade (SISBIO license no. 21811–1).

### DNA extraction and sequencing

The samples were separately ground in liquid nitrogen using a mortar and pestle, and the DNA was extracted with a 2% hexadecyl-trimethyl-ammonium bromide (CTAB) extraction buffer (pH 8.0) [0.5 M EDTA, 1 M Tris-HCl, 5 M NaCl, 2% CTAB], followed by a Chloroform:Isoamyl Alcohol (24:1, v/v) step. The disaggregated material was scraped into 2.0 mL microtubes containing preheated (65°C) extraction buffer at a 1:5 ratio (0.5 mL). RNAse (10 mg mL^–1^, Sigma-Aldrich, St Louis, MO, USA) was added, and the tubes were incubated for 20 min at 55°C. Next, Proteinase K (10 mg mL^–1^, Sigma-Aldrich, St Louis, MO, USA) was added, and the identical incubation conditions were provided as above. An equal volume of Chloroform:Isoamyl Alcohol mixture (24:1) was added to the extract and mixed by gentle inversion for 5 to 10 min to form a uniform emulsion. The mixture was centrifuged at 8,000 rpm for 10 min at room temperature. The aqueous phase was pipetted out gently, avoiding the interface. To the above solution, 3 M NaOAc and 0.6 of the total solution volume of cold isopropanol (-20°C) was added, and the tubes were incubated at -4°C for 20 min. The mixture was then centrifuged at 10,000 rpm for 30 min. The pellet was washed (2x) with 70% cold ethanol (-20°C), dried at room temperature, resuspended in 50 μL of TE buffer [10 mM Tris-HCl, 1 mM EDTA, pH 8.0], and stored at −20°C. DNA extraction from the sample was carried out in duplicate. The solutions and buffers were autoclaved at 121°C at 15 psi. The stock solutions of 10 mg mL^–1^ of RNase and Proteinase K were prepared according to the user’s manual. The integrity of the DNA samples was evaluated using electrophoresis on 1% agarose gels with GelRed^TM^ (Biotium Inc., Hayward, CA) to verify its quality, and the DNA purity was assessed with a NanoDrop spectrophotometer (Thermo Fisher Scientific Inc., Waltham, MA, USA). Accurate DNA quantification was obtained using a Qubit® 3.0 Fluorometer (Life Technologies-Invitrogen, Carlsbad, CA, USA). The DNA libraries were generated using a Nextera XT DNA Sample Preparation Kit (Illumina, San Diego, CA, USA). The size distribution of the libraries was evaluated using a 2100 Bioanalyzer (Agilent, Santa Clara, CA, USA), and DNA quantification was obtained using 7500 Real Time PCR (Applied Biosystems, Foster City, CA, USA) and KAPA Library Quantification Kits (Kapa Biosystems, Wilmington, MA, USA). Paired-end sequencing (2 × 250 bp) was performed on a MiSeq machine (Illumina, San Diego, CA, USA).

### Bioinformatics and statistical analysis of metagenomes

The fastq files generated by Illumina sequencing were qualitatively evaluated using FASTQC (v.0.11.2, http://www.bioinformatics.babraham.ac.uk/projects/fastqc/) [32]. The sequences were preprocessed with PRINSEQ (v0.20.4, http://edwards.sdsu.edu/cgi-bin/prinseq/prinseq.cgi) [33] to remove low quality DNA sequences (Phred score < 30), duplicates, and short sequences (< 35 bp). Paired-ended Illumina reads were merged using She-Ra software with default parameters and a quality metric score of 0.5 [34]. Sequence annotation was conducted via Metagenome Rapid Annotation using the Subsystem Technology (MG-RAST) webserver (http://metagenomics.nmpdr.org/) [35], using the following cut-off parameters: e-value lower of 1e^-5^, 60% of minimum sequence identity and at least 15 bp alignment length. Taxonomic annotation was performed using the GenBank database, (http://www.ncbi.nlm.nih.gov/) and functional annotation was completed using the SEED database [36]. The statistical analyses were performed with R version 3.0.3 [37], except where indicated. The abundance and multivariate figures were plotted with the ggplot2 and reshape packages [38], [39]. To test the hypotheses that the taxonomic (H1) and functional (H2) composition of the turf are conserved in space and time, and that the abundance of key genes (e.g., photosynthesis and chemosynthesis) are different among turf and other benthic holobionts (corals and rhodoliths) and seawater (H3), Permutational Multivariate Analysis of Variance (PERMANOVA) was performed using the “adonis” function of Vegan package [40] (Bray-Curtis distances and 999 permutations).

A collection of 22 metagenomes corresponding to corals, rhodoliths and seawater were retrieved from MG-RAST: eight metagenomes from coral (healthy and diseased) [41], six from rhodolith [42], [43], and eight from seawater [14] (Table 1). All the metagenome samples were from the Abrolhos Bank (Table 1) and were annotated with same databases to diminish possible annotation biases.

**Table 1:** General features of each metagenomic sample of the benthic organisms (turf, coral, and rhodolith) and seawater collected from the Abrolhos Bank. The metadata were retrieved from a MG-RAST server framework. The details of each collected sample are provided. The sampling reef sites are indicated, as well as their exact locations (latitude and longitude). The sampling depth (meters) and the sequencing technology used for each metagenome are listed. The total (bp) and read sizes given for the metagenomes are post quality-control values.

MG-RAST ID	Sample Name	Sample Type / Coral Disease Status	Local	Reef	PMID	Sampling date	Depth (m)	Lat.	Lon.	Technology of Sequencing	Size (reads)	Size (bp)
4561212.3	PL.1|M	Turf	Abrolhos Reefs	Pedra de Leste	this study	10/03/2013	12	-17.783	-39.051	Illumina/Solexa	172,944	31,448,043
4561207.3	PL.2|M	Turf	Abrolhos Reefs	Pedra de Leste	this study	10/03/2013	12	-17.783	-39.051	Illumina/Solexa	29,495	5,111,519
4561206.3	PL.3|M	Turf	Abrolhos Reefs	Pedra de Leste	this study	10/03/2013	12	-17.783	-39.051	Illumina/Solexa	554,519	95,157,293
4561211.3	PL.4|M	Turf	Abrolhos Reefs	Pedra de Leste	this study	10/03/2013	12	-17.783	-39.051	Illumina/Solexa	198,979	35,519,165
4561210.3	AR.1|M	Turf	Abrolhos Reefs	Archipelago (Mato Verde)	this study	10/03/2013	12	-17.964	-38.702	Illumina/Solexa	262,886	46,552,396
4561205.3	AR.2|M	Turf	Abrolhos Reefs	Archipelago (Mato Verde)	this study	10/03/2013	12	-17.964	-38.702	Illumina/Solexa	280,308	51,419,363
4561203.3	AR.3|M	Turf	Abrolhos Reefs	Archipelago (Mato Verde)	this study	10/03/2013	12	-17.964	-38.702	Illumina/Solexa	659,796	168,993,630
4561213.3	AR.4|M	Turf	Abrolhos Reefs	Archipelago (Mato Verde)	this study	10/03/2013	12	-17.964	-38.702	Illumina/Solexa	575,503	99,195,357
4561208.3	PAB.2|M	Turf	Abrolhos Reefs	Parcel dos Abrolhos	this study	10/03/2013	12	-17.998	-38.671	Illumina/Solexa	80,324	14,650,440
4561202.3	PAB.3|M	Turf	Abrolhos Reefs	Parcel dos Abrolhos	this study	10/03/2013	12	-17.998	-38.671	Illumina/Solexa	102,782	26,888,594
4561209.3	PAB.4|M	Turf	Abrolhos Reefs	Parcel dos Abrolhos	this study	10/03/2013	12	-17.998	-38.671	Illumina/Solexa	176,395	32,257,528
4564639.3	PL.2|O	Turf	Abrolhos Reefs	Pedra de Leste	this study	15/10/2013	12	-17.783	-39.051	Illumina/Solexa	107,665	18,350,484
4564642.3	PL.4|O	Turf	Abrolhos Reefs	Pedra de Leste	this study	15/10/2013	12	-17.783	-39.051	Illumina/Solexa	186,167	33,060,641
4564647.3	AR.1|O	Turf	Abrolhos Reefs	Archipelago (Portinho Norte)	this study	15/10/2013	12	-17.959	-38.701	Illumina/Solexa	45,168	8,118,890
4564646.3	AR.2|O	Turf	Abrolhos Reefs	Archipelago (Portinho Norte)	this study	15/10/2013	12	-17.959	-38.701	Illumina/Solexa	624,907	152,157,722
4564644.3	AR.3|O	Turf	Abrolhos Reefs	Archipelago (Portinho Norte)	this study	15/10/2013	12	-17.959	-38.701	Illumina/Solexa	249,663	74,875,522
4564648.3	PAB.1|O	Turf	Abrolhos Reefs	Parcel dos Abrolhos	this study	15/10/2013	12	-17.998	-38.671	Illumina/Solexa	535,455	134,542,669
4564643.3	PAB.2|O	Turf	Abrolhos Reefs	Parcel dos Abrolhos	this study	15/10/2013	12	-17.998	-38.671	Illumina/Solexa	151,800	29,152,445
4564645.3	PAB.3|O	Turf	Abrolhos Reefs	Parcel dos Abrolhos	this study	15/10/2013	12	-17.998	-38.671	Illumina/Solexa	586,084	146,060,007
4447483.3	California	Water	Abrolhos Reefs	California	22679480	29/01/2009	12	-18.102	-38.591	454/Roche	167,513	74,889,416
4447551.3	PAB5	Water	Abrolhos Reefs	Parcel dos Abrolhos	22679480	28/01/2009	20	-17.959	-38.506	454/Roche	126,741	53,914,839
4447862.3	Timbebas	Water	Abrolhos Reefs	Timbebas	22679480	27/01/2009	5.6	-17.478	-39.028	454/Roche	149,734	58,969,826
4448427.3	Sebastião Gomes	Water	Abrolhos Reefs	Sebastião Gomes	22679480	26/01/2009	4.7	-17.912	-39.129	454/Roche	10,906	3,768,178
4453304.3	Timbebas 2010	Water	Abrolhos Reefs	Timbebas	22679480	26/02/2010	3.2	-17.478	-39.028	454/Roche	67,439	27,057,181
4453305.3	Pedra do Leste 2010	Water	Abrolhos Reefs	Pedra de Leste	22679480	27/02/2010	4.7	-17.784	-39.051	454/Roche	31,365	13,219,777
4453371.3	PAB5 2010	Water	Abrolhos Reefs	Parcel dos Abrolhos	22679480	23/02/2010	2.3	-17.959	-38.506	454/Roche	79,476	29,639,075
4453372.3	Sebastião Gomes 2010	Water	Abrolhos Reefs	Sebastião Gomes	22679480	23/02/2010	5.6	-17.912	-39.129	454/Roche	39,792	11,785,037
4477768.3	R1	Rhodolith	Abrolhos Buracas	Buracas	23985749	12/7/2010	27	-17.914	-37.909	454/Roche	17,863	6,729,554
4477767.3	R2	Rhodolith	Abrolhos Buracas	Buracas	23985749	12/7/2010	27	-17.914	-37.909	454/Roche	27,229	10,794,633
4477763.3	R3	Rhodolith	Abrolhos Buracas	Buracas	23985749	12/6/2010	43	-17.856	-38.12	454/Roche	32,488	11,605,525
4477766.3	R4	Rhodolith	Abrolhos Buracas	Buracas	23985749	12/6/2010	43	-17.856	-38.12	454/Roche	18,171	6,999,671
4478209.3	R5	Rhodolith	Abrolhos Buracas	Buracas	23985749	12/6/2010	43	-17.856	-38.12	454/Roche	29,791	10,856,929
4477765.3	R6	Rhodolith	Abrolhos Buracas	Buracas	23985749	12/7/2010	51	-17.914	-37.909	454/Roche	19,207	6,633,451
4463374.3	SGS2	Coral / Healthy	Abrolhos Reefs	Sebastião Gomes	23314124	23/02/2010	3.2	-17.912	-39.129	454/Roche	27,275	10,566,518
4462249.3	GiSGW2	Coral / Diseased	Abrolhos Reefs	Sebastião Gomes	23314124	23/02/2010	3.2	-17.912	-39.129	454/Roche	20,483	7,615,646
4463363.3	SGW5	Coral / Diseased	Abrolhos Reefs	Sebastião Gomes	23314124	23/02/2010	3.2	-17.912	-39.129	454/Roche	29,048	11,075,785
4463368.3	P5S2	Coral / Healthy	Abrolhos Reefs	Parcel dos Abrolhos	23314124	23/02/2010	3.2	-17.959	-38.506	454/Roche	40,671	16,401,293
4463367.3	P5S4	Coral / Healthy	Abrolhos Reefs	Parcel dos Abrolhos	23314124	23/02/2010	5.6	-17.959	-38.506	454/Roche	11,949	4,745,703
4463366.3	P5S5	Coral / Healthy	Abrolhos Reefs	Parcel dos Abrolhos	23314124	23/02/2010	5.6	-17.959	-38.506	454/Roche	26,551	10,563,130
4463359.3	P5W2	Coral / Diseased	Abrolhos Reefs	Parcel dos Abrolhos	23314124	23/02/2010	5.6	-17.959	-38.506	454/Roche	7,406	2,725,373
4463358.3	P5W5	Coral / Diseased	Abrolhos Reefs	Parcel dos Abrolhos	23314124	23/02/2010	5.6	-17.959	-38.506	454/Roche	17,351	6,328,106

To verify if the samples from turf, rhodoliths, corals and seawater would group together according to their most important (see below) metagenomic (taxonomic and functional) features, a principal component analysis (PCA) was performed, using the “rda” function of Vegan package [40]. The goal of PCA is to explain as much of the variance as possible in the first few components, thus reducing the complexity of the data by combining related variables. We used the supervised Random Forest algorithm to select the variables (bacterial orders and subsystems) based on the “Mean Decreasing Accuracy” values [44] using the “randomForest” function of Random Forest R package [45]. The Variable Importance Measure Metric allows us to identify the most important variables for discriminating sample groups [46]. There are some approaches to estimate variables importance in separating samples into groups in supervised Random Forests. We have used mean decrease in accuracy to select the most important variables, which is determined in the error-calculating phase [46]. The purity of a node in a Random Forest is measured by the Gini index, so the mean decrease in Gini is a measure of variable contribution to the nodes and leaves homogeneity in the Random Forest. Random Forests are a robust approach for clustering metagenomes and metabolic processes in microbial communities from different environments [46]. To standardize the metagenome sizes, we present the metagenomic data as relative abundances (the number of sequences of a given taxa or subsystem of a metagenome divided by the total number of annotated sequences of this metagenome). Percentage data were transformed to arcsin(√x) for multivariate analysis. P-values of <0.05 were considered statistically significant.

### Analysis of pigments and ash-free dry weights

To assess the composition of the photosynthetic components of the turf, we quantified the pigments and determined their ash-free dry weights in the turf samples collected in October, 2013. Chlorophylls *a*, *b* and *c*_1+2_ (Chl*a*, Chl*b*, and Chl*c*, respectively), Pheophytin *a* (Pheo*a*), and Phycobiliproteins (PBPs) were measured. Chl*a*, Chl*b*, Chl*c*, and Pheo*a* were measured via spectrofluorometry using duplicate 0.2 g aliquots of turf material per sample. The pigments were extracted in 90% acetone:water. The cells were ruptured by grinding the material with a glass rod in the bottom of a glass test tube. The tubes were stored overnight at 4°C in the dark. After centrifugation, the fluorescence properties of the acetonic extracts were measured on a Varian Cary Eclipse® (wavelength accuracy ± 1.0 nm from 200–900 nm) spectrofluorometer (Agilent©, USA). The chlorophyll concentrations were assessed using a modified version of Neveux and Lantoine’s [47] method, which was described in [48]. Data acquisition was performed by recording the fluorescence emission spectra for each of the 31 excitation wavelengths (3 nm increments from 390–480 nm). The emission spectra were recorded at 2 nm intervals from 615–715 nm and yielded 51 points for each spectrum. The pigment concentrations were estimated from the resulting 1,581 data points. A least squares approximation technique was used to discard negative solutions.

For the quantification of the PBPs, the turf samples were suspended in 5 ml of 20 nM sodium acetate buffer, pH 5.5 (supplemented with 3 mM sodium azide and 10 mM disodium EDTA). Turf cells were ruptured via sonication intercalated with freeze and thaw cycles. The crude extracts were treated with 1% (w/v) streptomycin sulfate for 30 min at 4°C and centrifuged at 10,000 g for 10 min at 4°C to precipitate the cellular debris. The phycoerythrin, phycocyanin, and allophycocyanin concentrations were calculated from measurements of their optical densities at 564, 620, and 650 nm in a spectrophotometer. The quantification of the PBPs was based on the equations described by [49] and [50]. To obtain the ash-free dry weight (AFDW) of the turf samples, the material was dried at 60°C for 24 h, weighed, oxidized (ashed) in a muffle furnace at 450 ± 10°C for 4 h, cooled to room temperature in a desiccator, and then re-weighed.

## Results

### Morphological and pigment analyses

The turfs grew over the corals in the 19 samples collected at three locations. The turfs were phenotypically heterogeneous, exhibiting different colors (green, brown and red) and textures (S1 Fig). Microscopic analyses revealed that the turf assemblages were composed of filamentous, non-heterocystous cyanobacteria in a network with embedded seaweeds. Cyanobacteria were the structuring organisms in the turf, and morphotypes similar to the genera *Oscillatoria* and *Leptolyngbya* were commonly found. The minor turf components consisted of red (*Florideophyceae* members), green (filamentous *Bryopsis* sp. and flattened calcareous algae *Halimeda* sp., Ulvophyceae), and brown (fleshy *Dictyota* sp., Phaeophyceae) seaweeds. The Chlorophyll *a* per unit of turf area ranged from 7.2 (sample PAB.3|O) to 93.8 (sample PL.1|O) μg Chl*a* cm^-2^ (average 37.2; median 24.3 μg Chl*a* cm^-2^) (S1 Table).

Chlorophyll *b* accounted for a small fraction of the total chlorophyll concentration (average 8.5 μg Chl*b* cm^-2^). Chlorophyll *c*_*1+2*_ accounted for a minute amount of the total chlorophyll concentration (max. 3.2%). The AFDW of the turf samples averaged 0.29 mg cm^-2^, ranging from 0.11 (sample PAB.3|O) to 0.53 (sample PAB.1|O) mg cm^-2^. The Pheophytin *a*:Chlorophyll *a* ratio, a proxy for the degree of degradation in the photosynthetic community of the turf system, averaged 12.7%, ranging from 3.8% (representing the healthiest conditions) to 19.9% in the samples with the highest degree of chlorophyll degradation. Phycoerythrin was the main phycobiliprotein detected in the samples; its concentrations averaged 0.45 mg ml^-1^, with the maximum value found in the AR.3|O sample (1.9 mg ml^-1^), suggesting high abundance of cyanobacteria in turfs.

### Taxonomic assignment of turf metagenomic sequences

**Fig 1:**
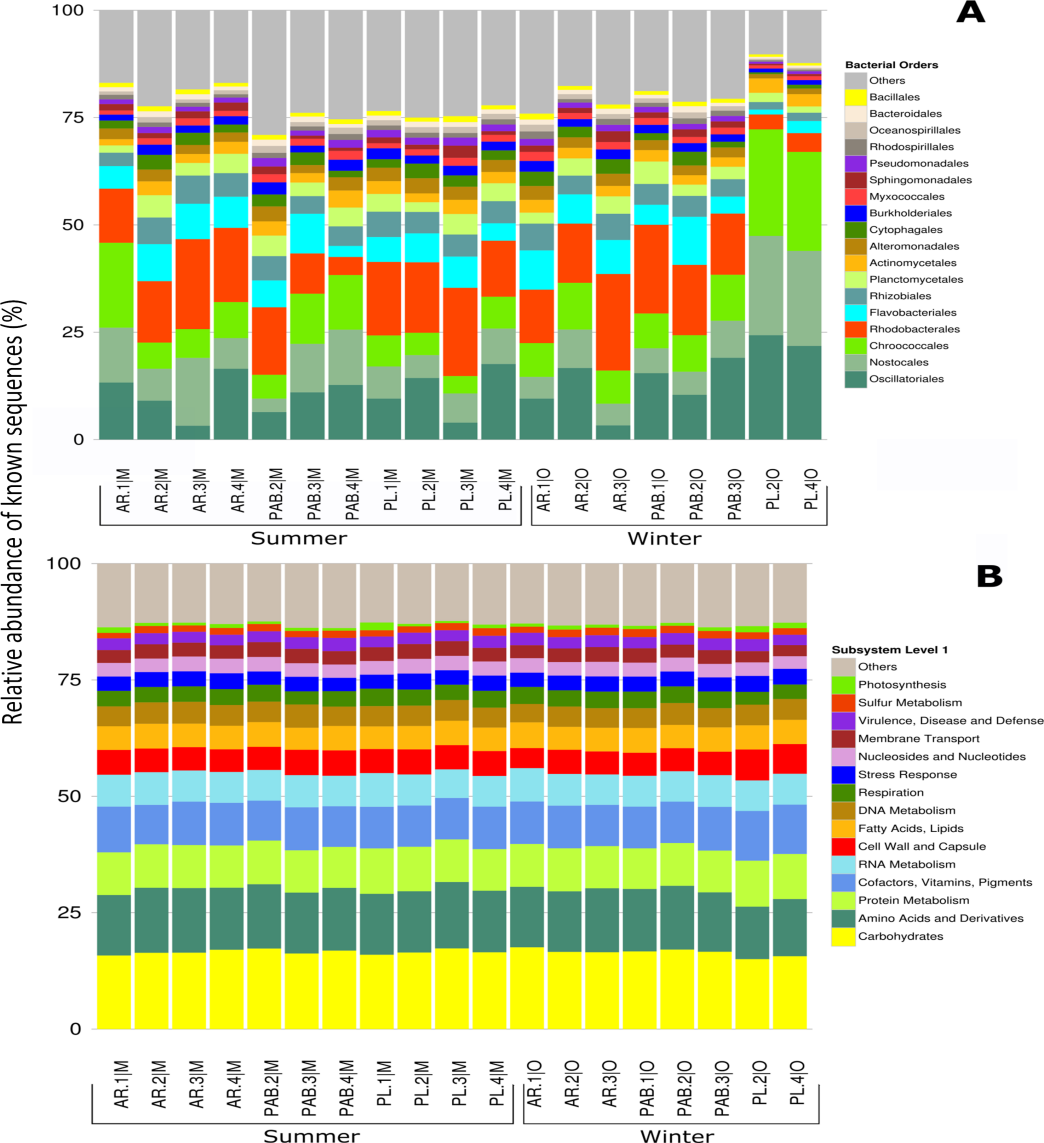
Turf system taxonomic and functional profiles. A) The major orders and B) subsystems (SEED Level 1) found in the metagenomes of the turf samples from the Abrolhos reefs. The percent correspond to the abundance normalized by the total of known sequences, i.e., genes with defined function. See Results section for the contribution of the major metabolisms. Sulfur metabolism accounted for 1.52% of the total metagenomes (± s.d. 0.14, N = 26,716), whereas nitrogen metabolism accounted for 1.13% (± s.d. 0.1, N = 19,939), and photosynthesis was responsible for 0.77% of the total metagenomes (± s.d. 0.3, N = 13,508). Ammonia assimilation related genes represented greater than 50% (52.3% ± s.d. 4.2) of the total genes in the nitrogen metabolism subsystems of the turf metagenomes, whereas N_2_ fixation genes represented 10.1% (± s.d. 5.7) of the total genes in this subsystem. Dissimilatory nitrate and nitrite ammonification corresponded to 18% ± s.d. 3.75 of the total genes in the nitrogen metabolism in this study). The sequences assigned as Clustering-based Subsystems (14.1%; N = 331,723 of the total) and Miscellaneous Subsystems (8.4%; N = 196,810 of total) were not included in the analysis.

The taxonomic assignments indicated that bacteria contributed an average of 85.92% (total N = 3,558,854) of the sequences annotated, ranging from 49.5% to 97.8%. Proteobacteria (40.57%, ± s.d. 10.36, N = 1.548,192), Cyanobacteria (35.04%, ± s.d. 15.5, N = 1.337,196), and Bacteroidetes (11.12%, ± s.d. 4.25, N = 424,185) were dominant in the turf microbiome (Fig 1A). Eukarya contributed 5.78% of the sequences on average (total N = 170,580), ranging from 1.45% to 42.11% of the total of all the metagenomes. Among the 19 samples, the macroalgae Chlorophyta (Chlorophyceae and Ulvophyceae), Pheophyta (Pheophyceae), and Rhodophyta (Florideophyceae) corresponded to, on average (% ± standard deviation), only 6.84 ± 5.7, 1.13 ± 1.12, and 1.09 ± 2.02, respectively, of the total Eukarya sequences. The Archaea domain and viruses contributed less than 1% of the sequences (a total of approximately 16,000 for Archaea and 2,000 for viruses). Unassigned and unclassified sequences corresponded to 7.81% of the average (total N = 315,994) (see S2 Table).

Turf samples had a statistically indistinguishable taxonomic profile at order level based on MG-RAST annotation, confirming the study hypothesis H1. No statistical difference in turf metagenomes composition in different locations and seasons were detected (Fig 1A and S3 Table).

The major taxa contributing to the core microbiome of these systems comprised typical aerobic anoxygenic photosynthesis (AANP) bacteria (e.g., Rhodobacteriales related to *Congregibacter*), Cyanobacteria (Oscillatoriales, Nostocales and Chroococcales), Flavobacteriales (related to *Maribacter*), Rhizobiales (related to *Roseobacter*, *Silicibacter* and *Dinoroseobacter*), and Planctomycetes (S4 Table).

The cyanobacterial sequences varied from approx. 19.8% (N = 7,036) in PL.3|M to approx. 72.25% (N = 58,906) in PL.2|O, whereas the Rhodobacterales sequences varied from approx. 4.4% (N = 3,383) in PL.2|O to approx. 22.5% (N = 37,695) in AR.3|O. The cyanobacteria sequences were related to *Trichodesmium* sp. (Oscillatoriales). Microscopic examination of the fixed samples (formaldehyde) revealed large quantities of cells that were phenotypically identified as *Leptolyngbya* sp., which is closely related to *Trichodesmium*.

### Metabolic diversity of turf metagenomes

According to the subsystems analysis, the turf metagenomes had a homogeneous profile, also confirming our hypothesis H2 (Fig 1B and S5 Table). The five most abundant subsystems (carbohydrates; amino acids and derivatives; protein metabolism; cofactors, vitamins, prosthetic groups, pigments; RNA metabolism) accounted for greater than 50% of all the classified metagenomic sequences (54.9% ± s.d. 3.5 for the five most abundant subsystems; N = 961,649 of the total) (see Fig 1B). Cofactors, vitamins, prosthetic groups and pigments made up the fourth most abundant subsystem; with the related folates and pterins (at level 2 of the classification according to the SEED database) being the fifth highest contributors (3.31% ± s.d. 0.17; N = 74,747), behind the level 2 core functions central carbohydrate metabolism, protein biosynthesis, and RNA processing and modification. Iron-sulfur metabolism involving the oxidative stress-related proteins of the YgfZ family (at SEED classification level 3) was abundant in all the metagenomes (March: N = 16,216; 1.5% ± s.d. 0.106 of all the metagenomes; October: N = 17,656; 1.5% ± s.d. 0.104 of all the metagenomes).

**Fig 2:**
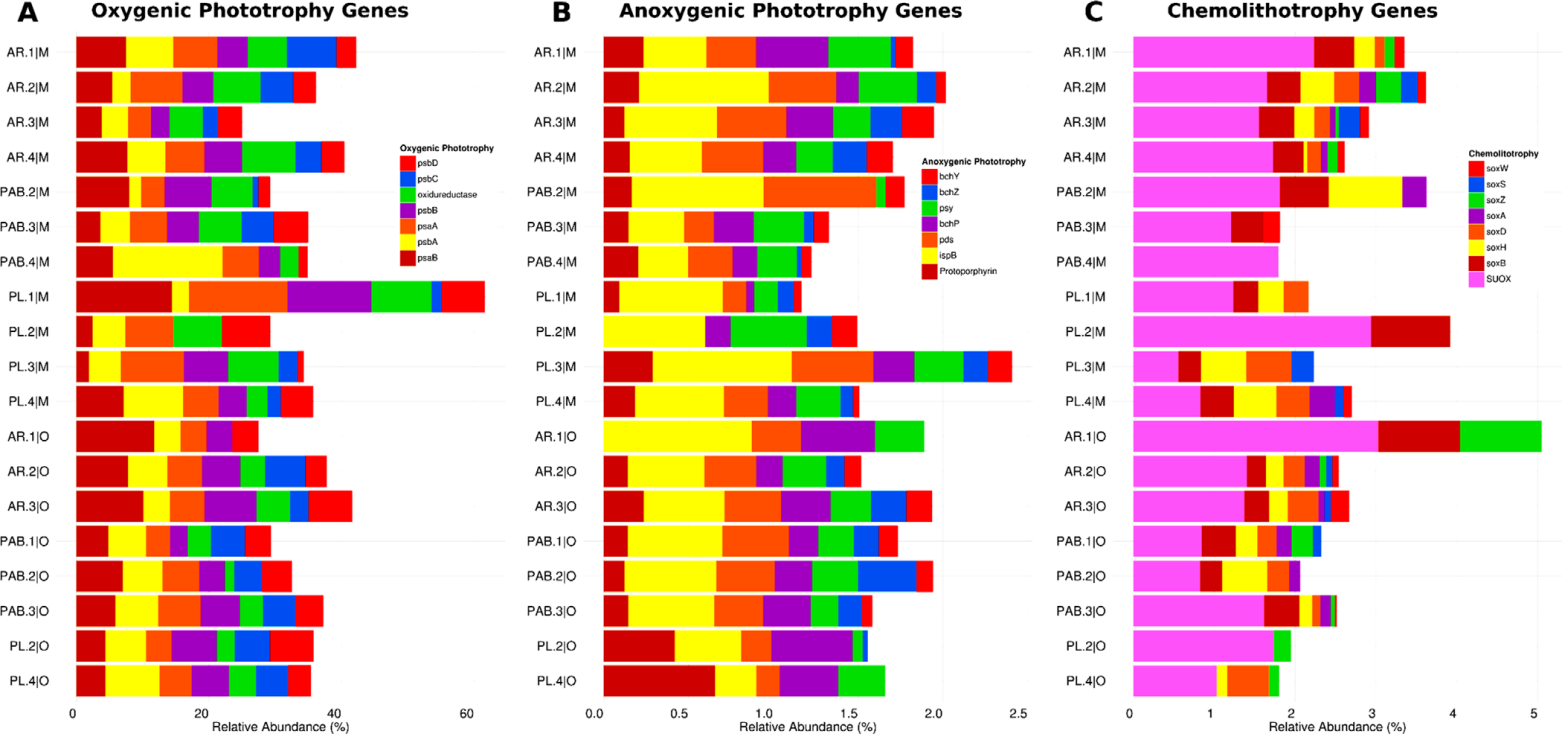
Most abundant genes from the different lifestyles co-occurring in Abrolhos Bank turfs. Genes involved in A) oxygenic photosynthesis, B) anoxygenic photosynthesis, and C) chemolithotrophy from each turf metagenome. Relative abundance of annotated sequences. Genes related to oxygenic photosynthesis are most abundant. The values in panel A varies from 0–60%; B from 0–2.5%, and C from 0–5%.

The photosynthesis subsystem had a total contribution ranging from 0.45% (PL.3|M) to 1.3% (PL.2|M) of the entire dataset. Approx. 46.7% (± s.d. 12.7), 33.82% (± s.d. 2.19), and 19.46% (± s.d. 1.74) of the sequences within the photosynthesis subsystem (N = 19,783) belonged to Cyanobacteria, Bacteria, and Eukarya, respectively. A total of 13,204 reads belonging to various chloroplast genes (i.e., rbc, pet, psa, psb, ndh, LSU, and SSU) were identified as Cyanobacteria (52% ± s.d. 37.01), Eukarya (45% ± s.d. 34.3), and Bacteria (3% ± s.d. 4.05). These chloroplast genes represent oxygenic phototrophs (Fig 2A), whereas other light harvesting genes (e.g., bchl, puf, and rhodopsin-based phototrophy genes, N = 6,969) indicated the presence of anoxygenic phototrophy in turfs (Fig 2B). Chemolithotrophic sulfur oxidation genes (i.e., sox genes, 1.9%, N = 739) were also present in the turfs (Fig 2C).

Sequences related to bacterial secretion systems at the functional level (within the subsystem membrane transport) corresponded to 13,638 out of 51,555 sequences and were identified as T1SS–Type 1 (1.16% of the total membrane transport subsystem, N = 623), T2SS (9.3%, N = 4,978), T3SS (0.9%, N = 460), T4SS (7.1%, N = 3,793), T5SS (0.15%, N = 84), and T6SS (6.9%, N = 3,700) secretion systems. T3SS, T4SS, T6SS interact directly with the host eukaryotic cell membrane, whereas T1SS, T2SS and T5SS secrete proteins into the extracellular milieu. The iron acquisition and metabolism subsystems (N = 18,561) included 6,758 sequences associated with iron acquisition in Vibrio (N = 7,660 sequences were identified as TonB-dependent receptors), 588 sequences of the siderophore pyoverdine (related to *Pseudomonas*), and 1,256 sequences related to iron acquisition *Streptococcus*. Sequences in the virulence, disease and defense subsystem corresponded to 1.7% (N = 1,219; PL.4|O) to 2.2% (N = 1,116; PAB.3|M) of the total metagenomes. Beta-lactamase resistance genes (N = 2,064, 4.9%), multidrug efflux pumps (N = 1,078, 2.5%), multidrug resistance efflux pumps (N = 4,760, 11.2%), and fluoroquinolone resistance genes (N = 6,034, 14.2%), at SEED classification level 3, represented approximately 31.8% of all the genes in the virulence subsystem. Superoxide dismutase (1,039 sequences) and sulfur metabolism sequences (at least 101 different genes, comprising 11 categories of assimilatory and dissimilatory pathways) were also discovered. The major metabolic features are depicted (S2 Fig).

### Turf microbiomes differ from the other microbiomes of the major Abrolhos benthic organisms

**Fig 3:**
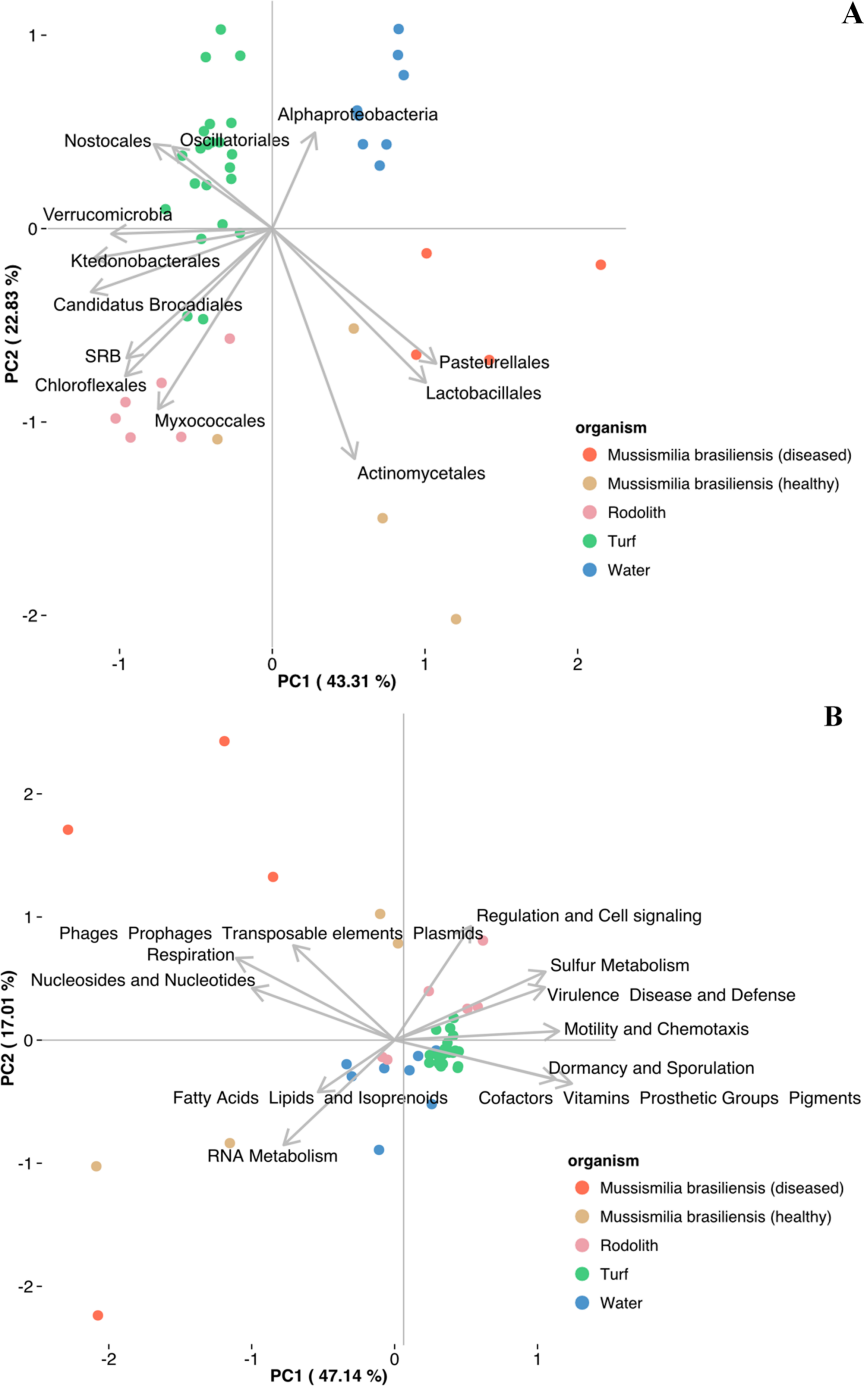
Principal Component analysis of the 41 metagenomes using the top eleven variables identified from the Random Forest analysis. Turf taxonomic and functional potential compared with other abundant benthic organisms and seawater from Abrolhos environments. A) The major orders and B) subsystems (SEED Level 1) found in the metagenomes of the turf samples from Abrolhos reefs compared with coral and rhodolith metagenomic data. Principal Component Analysis using 11 Level 1 SEED Subsystems selected based on the “Mean Decreasing Accuracy” of the Random Forest analysis. Metagenomic relative abundance data in percentages were transformed to arcsin(√x). Previously published seawater, coral, and rhodolith metagenome datasets were used in this analysis (Bruce et al., 2012; Garcia et al., 2013; Cavalcanti et al., 2013, 2014, respectively).

Turfs, corals, rhodoliths, and water formed different groups using PCA based on the most abundant taxa (Fig 3A) and functions (Fig 3B). All the turfs formed a tight group clearly separated from other abundant benthic organisms, also confirming our hypothesis H3. The two first axes explained a large proportion of the taxonomic variation between the samples (43.59% for PC1 and 23.02% for PC2) (Fig 3A). Nostocales and Oscillatoriales highly influenced the turf sample grouping (Fig 3A), in agreement with the RF results (S6 Table).

Likewise, the two first axes explained a large proportion of the functional variation between the samples (47.14% for PC1 and 17.01% for PC2) (Fig 3B). The following important functional features were found: virulence, disease, and defense; sulfur metabolism; motility and chemotaxis; dormancy and sporulation; cofactors, vitamins, prosthetic groups and pigments (Fig 3B, for RF results, see S7 Table). The number of nitrogen metabolism metagenomic sequences in the turfs was higher than those of the corals, rhodoliths, and seawater from Abrolhos (S8 Table).

Additionally, ANOVA results showed that the abundance of genes involved in oxygenic photosynthesis, anoxygenic photosynthesis and chemosythesis (Fig 2) are different among turfs and other benthic holobionts (corals and rhodoliths) and seawater (Table 2 and S9 Table).

**Table 2:** The abundance of genes related to oxygenic and anoxygenic photosynthesis and chemosynthesis are different among turf and other benthic holobionts (corals and rhodoliths) and seawater (H3). ANOVA results of total bacterial abundance. DF, degrees of freedom; SS, sum of squares; MS, mean sum of squares. Bonferroni method was used to adjust *p* values.

	DF	SS	MS	F value	*P* value	*P* value (adjusted)
**Oxygenic**						
Model	3	4572	1524.1	6.705	0.001	0.003
Residuals	37	8411	227.3			
**Anoxygenic**						
Model	3	31.950	10.651	4.875	0.006	0.018
Residuals	37	80.840	2.185			
**Chemolithotrophy**						
Model	3	10974	3658	16.26	<0.001	<0.001
Residuals	37	8323	225			

## Discussion

This study highlights the taxonomic and functional metagenomic consistency of turf as the most dominant benthic component of the Abrolhos reefs. Our hypotheses (H1 and H2) that turf have homogeneous microbial and functional compositions across space and time were confirmed. We also confirmed our hypothesis H3 as turfs had particular sets of genes involved in oxygenic and anoxygenic phothosynthesis and chemosynthesis not present in other benthic organisms (corals and rhodoliths) and seawater. The functional properties of the turf metagenomes hint a stable microbiome association. The association of cyanobacteria with aerobic, anaerobic, and AANP bacteria may increase turf stability and their competitive advantage (photosynthesis, nitrogen, and sulfur metabolism) in comparison with other autotrophic benthic components, such as corals and rhodoliths. The unexpected homogeneity in both taxonomy and function, despite their diversity in geographical origin, sampling time, biomass per unit of area, and pigments profile of their photosynthetic component (i.e., chlorophylls and phycoerythrin concentrations and pheophytin:Chl*a* ratios), corroborates this potential stable association. Furthermore, the metagenomic profiles of these systems clearly differed from those of other Abrolhos benthic organisms, which points toward the existence of a specific turf assemblage. The core microbiome of the turf consists of an assemblage of primary producers (both phototrophic and lithotrophic) and heterotrophic microbes possibly bound by complementary synergistic functions.

Photosynthesis in turfs

Oxygenic (by cyanobacteria and algae) and anoxygenic photosynthesis (e.g., by certain Rhodobacterales, Rhizobiales, and Rhodospirillales), sulfide production from sulfate reduction (by sulfate-reducing bacteria, SRB), and microbial sulfide oxidation overlap and fluctuate by day and night, during which sulfate-reducing bacteria in the surface layer must tolerate oxygen exposure as a result of cyanobacterial photosynthesis during the day [51]. The co-occurrence of different photosynthetic organisms in turf allows them to use light under the typically diverse environmental conditions (i.e., high and low luminous incidence and water turbidity) of reef systems. Eukaryotic photosynthetic algae and cyanobacteria possess Chlorophyll *a*, which permits light capture at both 430 nm (blue light) and 680 nm (red light). Nevertheless, cyanobacteria utilize phycobilisomes (large pigment-protein complexes) to capture photons between the blue and red regions of the spectrum, which are not efficiently trapped by chlorophyll [52]. On the other hand, AANP bacteria possess bacteriochlorophyll, which allows for light harvest at ultraviolet (360 nm) and infrared wavelengths (805 nm and 870 nm). In general, zooxanthellate corals such as *Orbicella*, *Mussismilia*, and *Porites* have a narrower light usage range because they rely upon Chlorophyll *a* containing phototrophs (e.g., *Symbiodinium* and cyanobacteria) [53]. However, endolytic chlorophyll *d*-containing cyanobacteria have been found in association with dead corals [54]. Chlorophyll *d* allows for near infrared (700–740 nm) light harvesting and photosynthesis [55]. Large quantities of O_2_ can be produced by turfs. Bacterial persistence in these systems is enabled through the production of enzymes (e.g., superoxide dismutase) that avoid oxidative stress by neutralizing reactive oxygen species such as hydrogen peroxide, organic peroxides, and superoxide [6], [56], [57].

Sulfur cycle in turfs

Sulfate-reducing bacteria (e.g., *Desulfovibrio*, *Desulfobacterium*, and *Desulfobulbus*) have adapted to the oxic conditions of turf surface layers through motility and aggregate formation [51]. The sulfide produced by these bacteria as a respiration product can be utilized by AANP bacteria and cyanobacteria. The latter is commonly found in assemblages with continuous exposure to biogenically produced sulfide, in which fluctuations of anaerobic and aerobic conditions are common [58]. We have shown that turf microbiomes are rich in anaerobic and facultative anaerobic microbes, which corroborates previous studies [7]. Sulfide acts as an electron donor to anoxygenic photosynthesis in *Rhodobacteraceae* members and cyanobacteria. Additionally, sulfide serves as an assimilatory sulfur source and is highly toxic, reacting with various cytochromes, hemoproteins, and other compounds. These reactions inhibit the electron transport chain, blocking respiration as well as oxygenic and anoxygenic photosynthesis. The capacity of turfs to engage in S-cycle reactions indicates their competence to thrive under harsh conditions. Coral reefs provide a habitat enriched in dissolved and particulate organic material, such as amino acids, sugars and dimethylsulfoniopropionate (DMSP), which trigger the development of DMSP-degrading bacteria in turfs. Organic sulfur compounds, particularly DMSP and gas dimethyl sulfide (DMS), are important for the structuring of coral and turf-associated bacterial communities [1], [59], [60]. The present study indicates that sulfur metabolism is an important factor for distinguishing Abrolhos turfs from corals and rhodoliths. In contrast with corals, turfs tolerate higher levels of sulfide, which is a consequence of their core microbiome [58], [61].

Turfs appear to be more stable to environmental changes (e.g., nutrients and temperature) and display higher growth rates and metabolic potential than other reef organisms (e.g., corals and rhodoliths) [62]. Turfs can grow faster than calcifying organisms, broadly occupying benthic habitats, particularly outside of the National Abrolhos Marine Park areas, while corals and rhodoliths grow only a few millimeters per year [63]. In addition, turfs can obtain limited nutrients in the environment for growth, e.g., iron, through secreted bacterial siderophores [64], [65–67]. Antimicrobial production and antimicrobial resistance can modulate the homeostasis of microbial populations because they are also involved in scavenging and the uptake of nutrients [65].

Phototrophic organisms, represented by non-heterocystous and heterocystous cyanobacteria, are the most abundant components of turfs, suggesting that they play an important role in the nitrogen cycle of Abrolhos reef systems. Indeed, the number of nitrogen metabolism metagenomic sequences in the turfs was higher than those of the corals, rhodoliths, and seawater from Abrolhos. Ammonia assimilation related genes represented greater than 50% (52.3% ± s.d. 4.2) of the total genes in the nitrogen metabolism subsystems of the turf metagenomes, whereas N_2_ fixation genes represented 10.1% (± s.d. 5.7) of the total genes in this subsystem. Ammonia can be produced in turfs via dissimilatory nitrate and nitrite ammonification (18% ± s.d. 3.75 of the total genes in the nitrogen metabolism in this study), a process carried out by the sulfate-reducing bacteria (such as *Desulfovibrio*, *Desulfobacterium*, and *Desulfobulbus*) found in this study and several types of Proteobacteria [68].

## Conclusion

The turfs of the Abrolhos Bank have characteristic microbial communities and conserved metabolic profiles revealed by metagenomics, particularly regarding the coupling between oxygenic photosynthesis, AANP, ammonia assimilation, N_2_ fixation, and the S cycle. The turf microbiome is involved in the core functions of the turfs and may promote the proliferation of this dominant benthic component in the Abrolhos Bank.

## DNA Deposition

All metagenomic data are held at http://metagenomics.anl.gov (Metagenomics RAST Server), according the following MG-RAST IDs: 4561212.3, 4561207.3, 4561206.3, 4561211.3, 4561210.3, 4561205.3, 4561203.3, 4561213.3, 4561208.3, 4561202.3, 4561209.3, 4564639.3, 4564642.3, 4564647.3, 4564646.3, 4564644.3, 4564648.3, 4564643.3, 4564645.3; and fasta files are available at https://marinebiodiversity.lncc.br/files/index.php/s/3HqRMATuGDQWDU6 (fthompson.6.1) (Brazilian Marine Biodiversity Database, BaMBa).

## Supporting Information

S1 Fig Underwater pictures of turfs. A, B, C, turf growing over *Mussismilia* corals. D, turf growing over *Orbicella* coral.
(TIF)

S2 Fig Scheme of turf system and major metabolisms. Conceptual model presenting the major metabolisms acting in turf from Abrolhos reefs, which are not a common function in other holobionts (e.g., sulphate reduction and anoxygenic photosynthesis absent in healthy corals).
(TIF)

S1 Table Turf pigment profile. Pigment concentration relative to area in the samples of turf assemblages collected in October 2013, ordered by hierarchical clustering. Chl: Chlorophyll; Phae: Phaeophytin; AFDW: ash-free dry weight.
(XLSX)

S2 Table General features of the turf assemblage metagenomes. Approximately 7 Gbp and 11.63 million reads (raw sequences) were obtained from turfs at different locations of the Abrolhos Bank. Approximately 6.5 million reads (11 samples) and 5.13 million reads (eight samples) were obtained from the turfs collected in March (summer) and October (winter) 2013, respectively. After quality control, the metagenomes contained 58,990 to 1,172,168 reads, with an average read length of 162 to 344 nt. The metagenomic sequences had high taxonomic coverage according to the rarefaction curve.
(XLSX)

S3 Table The taxonomic composition of turf metagenomes are statistically indistinguishable in different locations and seasons (H1). Adonis (PERMANOVA) results of taxonomic composition of turf metagenomes (bacterial order level) based on Bray-Curtis distances with 999 permutations. MS, mean sum of squares; SS, sum of squares. D.f., degrees of freedom; SS, sum of squares; MS, mean sum of squares.
(DOCX)

S4 Table Taxonomic contribution to the turf composition from the Abrolhos Bank, indicated by collection period and reef location. The percent correspond to the abundance normalized by the total of known sequences A) Contributions of the major phyla in the composition of turf metagenomes. B) Proteobacteria and cyanobacteria: Contributions of the most abundant closest species identified of Proteobacteria and cyanobacteria in each reef and collection period. C) Contributions of archaea and viruses (closest species) in each reef and collection period from the Abrolhos Bank.
(XLSX)

S5 Table The functional composition of turf metagenomes are statistically indistinguishable in different locations and seasons (H2). Adonis (PERMANOVA) results of functional composition of turf metagenomes (SEED Level 1 Subsystems) based on Bray-Curtis distances with 999 permutations. MS, mean sum of squares; SS, sum of squares. D.f., degrees of freedom; SS, sum of squares; MS, mean sum of squares.
(DOCX)

S6 Table Variable importance (taxonomic Order level) determined by the unsupervised Random Forest analysis ranking of Taxonomy. (XLSX)

S7 Table Variable importance (level 1 Subsystems) determined by the unsupervised Random Forest analysis ranking of Function. (XLSX)

S8 Table Comparison of the different types of metabolism in the turf, coral, rhodolith, and seawater samples from the Abrolhos Bank. The percentage, abundance, minimum, maximum, average and standard deviation are provided for each sample, as well as for photosynthesis, nitrogen, and sulfur metabolism.
(XLSX)

S9 Table Turkey Honest Significant Differences (HSD) *pos hoc* test results of H3 ANOVA. Diff., difference in the observed means.
(DOCX)

## References

[1] Barott K, Rodriguez-Brito B, Janouškovec J, Marhaver K, Smith J, Keeling, et al (2011) Microbial diversity associated with four functional groups of benthic reef algae and the reef-building coral Montastraea annularis. Environmental Microbiology 13: 1192–1204. 10.1111/j.1462-2920.2010.02419.x 21272183

[2] Barott K, Rodriguez-Mueller B, Youle M, Marhaver KL, Vermeij MJA, Smith JE, et al (2012) Microbial to reef scale interactions between the reef-building coral Montastraea annularis and benthic algae. Proc R Soc B 279, 1655–1664. 10.1098/rspb.2011.2155 22090385 PMC3282354

[3] Roff G, Mumby P (2012) Global disparity in the resilience of coral reefs. Trends in Ecology & Evolution 27: 404–413. 22658876 10.1016/j.tree.2012.04.007

[4] Sweet M, Burn D, Croquer A, Leary P (2013) Characterisation of the Bacterial and Fungal Communities Associated with Different Lesion Sizes of Dark Spot Syndrome Occurring in the Coral Stephanocoenia intersepta. PLoS ONE 8(4): e62580 10.1371/journal.pone.0062580 23630635 PMC3632600

[5] Egan S, Harder T, Burke C, Steinberg P, Kjelleberg S, Thomas T (2013) The seaweed holobiont: understanding seaweed-bacteria interactions. FEMS Microbiology Reviews 37: 462–476. 10.1111/1574-6976.12011 23157386

[6] Hollants J, Leliaert F, De Clerck O, Willems A (2013) What we can learn from sushi: a review on seaweed-bacterial associations. FEMS Microbiol Ecol 83: 1–16. 10.1111/j.1574-6941.2012.01446.x 22775757

[7] Hester E, Barott K, Nulton J, Vermeij M, Rohwer F (2016) Stable and sporadic symbiotic communities of coral and algal holobionts. The ISME Journal. 10(5):1157–69. 10.1038/ismej.2015.190 26555246 PMC5029208

[8] Goldberg WM (2013) The Biology of Reefs and Reef Organisms, University of Chicago Press.

[9] Sheppard CRC, Davy SK, Pilling GM (2009) The Biology of Coral Reefs (Biology of Habitats), OUP Oxford.

[10] Francini-Filho R, Coni E, Meirelles P, Amado-Filho G, Thompson F, Pereira-Filho G, et al (2013) Dynamics of Coral Reef Benthic Assemblages of the Abrolhos Bank, Eastern Brazil: Inferences on Natural and Anthropogenic Drivers. PLoS ONE 8: e54260 10.1371/journal.pone.0054260 23365655 PMC3554776

[11] Knowlton N, Jackson J (2008) Shifting Baselines, Local Impacts, and Global Change on Coral Reefs. PLoS Biology 6: e54 10.1371/journal.pbio.0060054 18303956 PMC2253644

[12] Dinsdale E, Edwards R, Hall D, Angly F, Breitbart M, Brulc J, et al (2008) Functional metagenomic profiling of nine biomes. Nature 455: 830–830. 10.1038/nature06810 18337718

[13] Sandin S, Smith J, DeMartini E, Dinsdale E, Donner S, Friedlander A, et al (2008) Baselines and Degradation of Coral Reefs in the Northern Line Islands. PLoS ONE 3: e1548 10.1371/journal.pone.0001548 18301734 PMC2244711

[14] Bruce T, Meirelles P, Garcia G, Paranhos R, Rezende C, de Moura R, et al (2012) Abrolhos Bank Reef Health Evaluated by Means of Water Quality, Microbial Diversity, Benthic Cover, and Fish Biomass Data. PLoS ONE 7: e36687 10.1371/journal.pone.0036687 22679480 PMC3367994

[15] Barott K, Williams G, Vermeij M, Harris J, Smith J, Rohwer F, et al (2012) Natural history of coral-algae competition across a gradient of human activity in the Line Islands. Marine Ecology Progress Series 460: 1–12.

[16] Barott K, Rohwer F (2012) Unseen players shape benthic competition on coral reefs. Trends in Microbiology 20: 621–628. 10.1016/j.tim.2012.08.004 22944243

[17] Haas A, Gregg A, Smith J, Abieri M, Hatay M, Rohwer F (2013) Visualization of oxygen distribution patterns caused by coral and algae. PeerJ 1: e106 10.7717/peerj.106 23882443 PMC3719126

[18] Haas A, Nelson C, Rohwer F, Wegley-Kelly L, Quistad S, Carlson C, et al (2013) Influence of coral and algal exudates on microbially mediated reef metabolism. PeerJ 1: e108 10.7717/peerj.108 23882445 PMC3719129

[19] Haas AF, Rohwer F, Carlson C, Nelson C, Wegley L, Leichter J, et al 2011 Effects of coral reef benthic primary producers on dissolved organic carbon and microbial activity. PLoS ONE 6:e27973 10.1371/journal.pone.0027973 22125645 PMC3220721

[20] Nelson CE, Goldberg SJ, Kelly LW, Haas AF, Smith JE, Rohwer F, et al 2013 Coral and macroalgal exudates vary in neutral sugar composition and differentially enrich reef bacterioplankton lineages. The ISME Journal 7:962–979. 10.1038/ismej.2012.161 23303369 PMC3635233

[21] Dinsdale EA, Pantos O, Smriga S, Edwards RA, Angly F, Wegley L, et al 2008 Microbial ecology of four coral atolls in the Northern Line Islands. PLoS ONE 3(2):e1584 10.1371/journal.pone.0001584 18301735 PMC2253183

[22] Rosenberg E, Koren O, Reshef L, Efrony R, Zilber-Rosenberg I (2007) The role of microorganisms in coral health, disease and evolution. Nature Reviews Microbiology 5: 355–362. 17384666 10.1038/nrmicro1635

[23] Casadevall A, Pirofski LA (2015) What is a host? Incorporating the microbiota into the damage-response framework. Infect Immun 83(1):2–7. 10.1128/IAI.02627-14 25385796 PMC4288903

[24] Moran NA, Sloan DB (2015) The Hologenome Concept: Helpful or Hollow? PLoS Biol 13(12): e1002311 10.1371/journal.pbio.1002311 26636661 PMC4670207

[25] Stal LJ (1995) Physiological ecology of cyanobacteria in microbial mats and other communities. New Phytol 131:1–32. 10.1111/j.1469-8137.1995.tb03051.x 33863161

[26] Connell S, Foster M, Airoldi L (2014) What are algal turfs? Towards a better description of turfs. Marine Ecology Progress Series 495: 299–307.

[27] Echenique-Subiabre I, Villeneuve A, Golubic S, Turquet J, Humbert J, Gugger M (2014) Influence of local and global environmental parameters on the composition of cyanobacterial mats in a tropical lagoon. Microb Ecol 69: 234–244. 10.1007/s00248-014-0496-0 25260923

[28] Ainsworth TD, Krause L, Bridge T, Torda G, Raina JB, Zakrzewski M, et al (2015) The coral core microbiome identifies rare bacterial taxa as ubiquitous endosymbionts. The ISME Journal 9 2261–2274, 10.1038/ismej.2015.39 25885563 PMC4579478

[29] Fierer N, Leff JW, Adams BJ, Nielsen UN, Bates ST, Lauber CL, et al (2012) Cross-biome metagenomic analyses of soil microbial communities and their functional attributes. Proc Natl Acad Sci USA 109 21390–21395. 10.1073/pnas.1215210110 23236140 PMC3535587

[30] Yatsunenko T, Rey FE, Manary MJ, Trehan I, Dominguez-Bello MG, Contreras M, et al (2012) Human gut microbiome viewed across age and geography. Nature 486(7402):222–227. 10.1038/nature11053 22699611 PMC3376388

[31] Mason O U, Hazen TC, Borglin S, Chain PS, Dubinsky EA, Fortney JL, et al (2012) Metagenome, metatranscriptome and single-cell sequencing reveal microbial response to Deepwater Horizon oil spill. ISME Journal 6:1715–1727. 10.1038/ismej.2012.59 22717885 PMC3498917

[32] Andrews S (2010) FastQC: a quality control tool for high throughput sequence data.

[33] Schmieder R, Edwards R (2011) Quality control and preprocessing of metagenomic datasets. Bioinformatics 27: 863–864. 10.1093/bioinformatics/btr026 21278185 PMC3051327

[34] Rodrigue S, Materna A, Timberlake S, Blackburn M, Malmstrom R, Alm E, et al (2010) Unlocking Short Read Sequencing for Metagenomics. PLoS ONE 5: e11840 10.1371/journal.pone.0011840 20676378 PMC2911387

[35] Meyer F, Paarmann D, D'Souza M, Olson R, Glass E, Kubal M, et al (2008) The metagenomics RAST server—a public resource for the automatic phylogenetic and functional analysis of metagenomes. BMC Bioinformatics 9: 386 10.1186/1471-2105-9-386 18803844 PMC2563014

[36] Overbeek R (2005) The Subsystems Approach to Genome Annotation and its Use in the Project to Annotate 1000 Genomes. Nucleic Acids Research 33: 5691–5702. 16214803 10.1093/nar/gki866 PMC1251668

[37] R Development Core Team (2011) R: A Language and Environment for Statistical Computing. Available: https://www.r-project.org/.

[38] Wickham H (2007) Reshaping Data with the reshape Package. Journal of Statistical Software 21(12):1–20.

[39] Wickham H (2009) ggplot2: elegant graphics for data analysis Springer Publishing Company.

[40] Oksanen J, Kindt R, O’Hara B (2005) Vegan: R functions for vegetation ecologists. Available: http://cc.oulu.fi/jarioksa/softhelp/vegan.html.

[41] Garcia G, Gregoracci G, de O Santos E, Meirelles P, Silva G, Edwards R, et al (2013) Metagenomic Analysis of Healthy and White Plague-Affected Mussismilia braziliensis Corals. Microb Ecol 65: 1076–1086. 10.1007/s00248-012-0161-4 23314124

[42] Cavalcanti G, Gregoracci G, Longo L, Bastos A, Ferreira C, Francini-Filho R, et al (2013) Sinkhole-like structures as bioproductivity hotspots in the Abrolhos Bank. Continental Shelf Research 70: 126–134.

[43] Cavalcanti G, Gregoracci G, dos Santos E, Silveira C, Meirelles P, Longo L, et al (2014) Physiologic and metagenomic attributes of the rhodoliths forming the largest CaCO3 bed in the South Atlantic Ocean. The ISME Journal 8: 52–62. 10.1038/ismej.2013.133 23985749 PMC3869012

[44] Breiman L (2001) Random Forests. Machine Learning 1–33.

[45] Liaw A, Wiener M (2002) Classification and regression by randomForest. R news 2:18–22.

[46] Dinsdale E (2013) Multivariate analysis of functional metagenomes. Front Gene 2 4:41. 10.3389/fgene.2013.00041 PMC3619665 23579547

[47] Neveux J, Lantoine F (1993) Spectrofluorometric assay of chlorophylls and phaeopigments using the least squares approximation technique. Deep Sea Research Part I: Oceanographic Research Papers 40: 1747–1765.

[48] Tenário M, Le Borgne R, Rodier M, Neveux J (2005) The impact of terrigeneous inputs on the Bay of Ouinné (New Caledonia) phytoplankton communities: A spectrofluorometric and microscopic approach. Estuarine, Coastal and Shelf Science 64: 531–545.

[49] Bennett A (1973) Complementary chromatic adaptation in a filamentous blue-green alga. The Journal of Cell Biology 58: 419–435. 4199659 10.1083/jcb.58.2.419 PMC2109051

[50] Bryant D, Guglielmi G, de Marsac N, Castets A, Cohen-Bazire G (1979) The structure of cyanobacterial phycobilisomes: a model. Archives of Microbiology 123: 113–127.

[51] Teske A, Ramsing NB, Habicht K, Fukui M, Küver J, Jørgensen BB, et al (1998) Sulfate-Reducing Bacteria and their activities in cyanobacterial mats of Solar Lake (Sinai, Egypt). Applied and Environmental Microbiology, 64(8), 2943–2951. 9687455 10.1128/aem.64.8.2943-2951.1998 PMC106797

[52] Steunou AS, Bhaya D, Bateson MM, Melendrez MC, Ward DM, Brecht E, et al (2006) In situ analysis of nitrogen fixation and metabolic switching in unicellular thermophilic cyanobacteria inhabiting hot spring microbial mats. Proc. Natl Acad. Sci. USA, 103: 2398–2403. 16467157 10.1073/pnas.0507513103 PMC1413695

[53] Lesser M (2004) Discovery of Symbiotic Nitrogen-Fixing Cyanobacteria in Corals. Science 305: 997–1000. 15310901 10.1126/science.1099128

[54] Behrendt L, Larkum A, Norman A, Qvortrup K, Chen M, Ralph P, et al (2010) Endolithic chlorophyll d-containing phototrophs. The ISME Journal 5: 1072–1076. 10.1038/ismej.2010.195 21160540 PMC3131860

[55] Chen M, Schliep M, Willows R, Cai Z, Neilan B, Scheer H (2010) A Red-Shifted Chlorophyll. Science 329: 1318–1319. 10.1126/science.1191127 20724585

[56] Egan S, Fernandes N, Kumar V, Gardiner M, Thomas T (2014) Bacterial pathogens, virulence mechanism and host defence in marine macroalgae. Environ Microbiol 16: 925–938. 10.1111/1462-2920.12288 24112830

[57] Fernandes N, Case R, Longford S, Seyedsayamdost M, Steinberg P, Kjelleberg S, et al (2011) Genomes and Virulence Factors of Novel Bacterial Pathogens Causing Bleaching Disease in the Marine Red Alga Delisea pulchra. PLoS ONE 6: e27387 10.1371/journal.pone.0027387 22162749 PMC3230580

[58] Richardson L, Miller A, Broderick E, Kaczmarsky L, Gantar M, Stanic D, et al (2009) Sulfide, microcystin, and the etiology of black band disease. Diseases of Aquatic Organisms 87: 79–90. 10.3354/dao02083 20095243 PMC3518071

[59] Raina J, Dinsdale E, Willis B, Bourne D (2010) Do the organic sulfur compounds DMSP and DMS drive coral microbial associations? Trends in Microbiology 18: 101–108. 10.1016/j.tim.2009.12.002 20045332

[60] Raina J, Tapiolas D, Forêt S, Lutz A, Abrego D, Ceh J, et al (2013) DMSP biosynthesis by an animal and its role in coral thermal stress response. Nature 502: 677–680. 10.1038/nature12677 24153189

[61] Weber M, de Beer D, Lott C, Polerecky L, Kohls K, Abed R, et al (2012) Mechanisms of damage to corals exposed to sedimentation. Proceedings of the National Academy of Sciences 109: E1558–E1567. 10.1073/pnas.1100715109 PMC3386076 22615403

[62] Welker M, Von Döhren H (2006) Cyanobacterial peptides—Nature's own combinatorial biosynthesis. FEMS Microbiology Reviews 30: 530–563. 16774586 10.1111/j.1574-6976.2006.00022.x

[63] Amado-Filho G, Moura R, Bastos A, Salgado L, Sumida P, Guth A, et al (2012) Rhodolith Beds Are Major CaCO3 Bio-Factories in the Tropical South West Atlantic. PLoS ONE 7: e35171. 22536356 10.1371/journal.pone.0035171 PMC3335062

[64] Cordero O, Ventouras L, DeLong E, Polz M (2012) Public good dynamics drive evolution of iron acquisition strategies in natural bacterioplankton populations. Proceedings of the National Academy of Sciences 109: 20059–20064. 10.1073/pnas.1213344109 PMC3523850 23169633

[65] Cordero O, Wildschutte H, Kirkup B, Proehl S, Ngo L, Hussain F, et al (2012) Ecological Populations of Bacteria Act as Socially Cohesive Units of Antibiotic Production and Resistance. Science 337: 1228–1231. 10.1126/science.1219385 22955834

[66] Costa T, Felisberto-Rodrigues C, Meir A, Prevost M, Redzej A, Trokter M, Waksman G (2015) Secretion systems in Gram-negative bacteria: structural and mechanistic insights. Nature Reviews Microbiology 13: 343–359. 10.1038/nrmicro3456 25978706

[67] Faraldo-Gómez J, Sansom M (2003) Acquisition of siderophores in Gram-negative bacteria. Nature Reviews Molecular Cell Biology 4: 105–116. 12563288 10.1038/nrm1015

[68] Yoon S, Cruz-García C, Sanford R, Ritalahti K, Löffler F (2014) Denitrification versus respiratory ammonification: environmental controls of two competing dissimilatory NO3−/NO2− reduction pathways in Shewanella loihica strain PV-4. The ISME Journal 9: 1093–1104. 10.1038/ismej.2014.201 25350157 PMC4409154

