# Supplementary figures and images for: Taxonomic and Functional Metagenomic Signature of Turfs in the Abrolhos Reef System (Brazil)
Source: PLoS One. 2016 Aug 22;11(8):e0161168. doi: 10.1371/journal.pone.0161168 (PMC4993507; doi:10.1371/journal.pone.0161168)

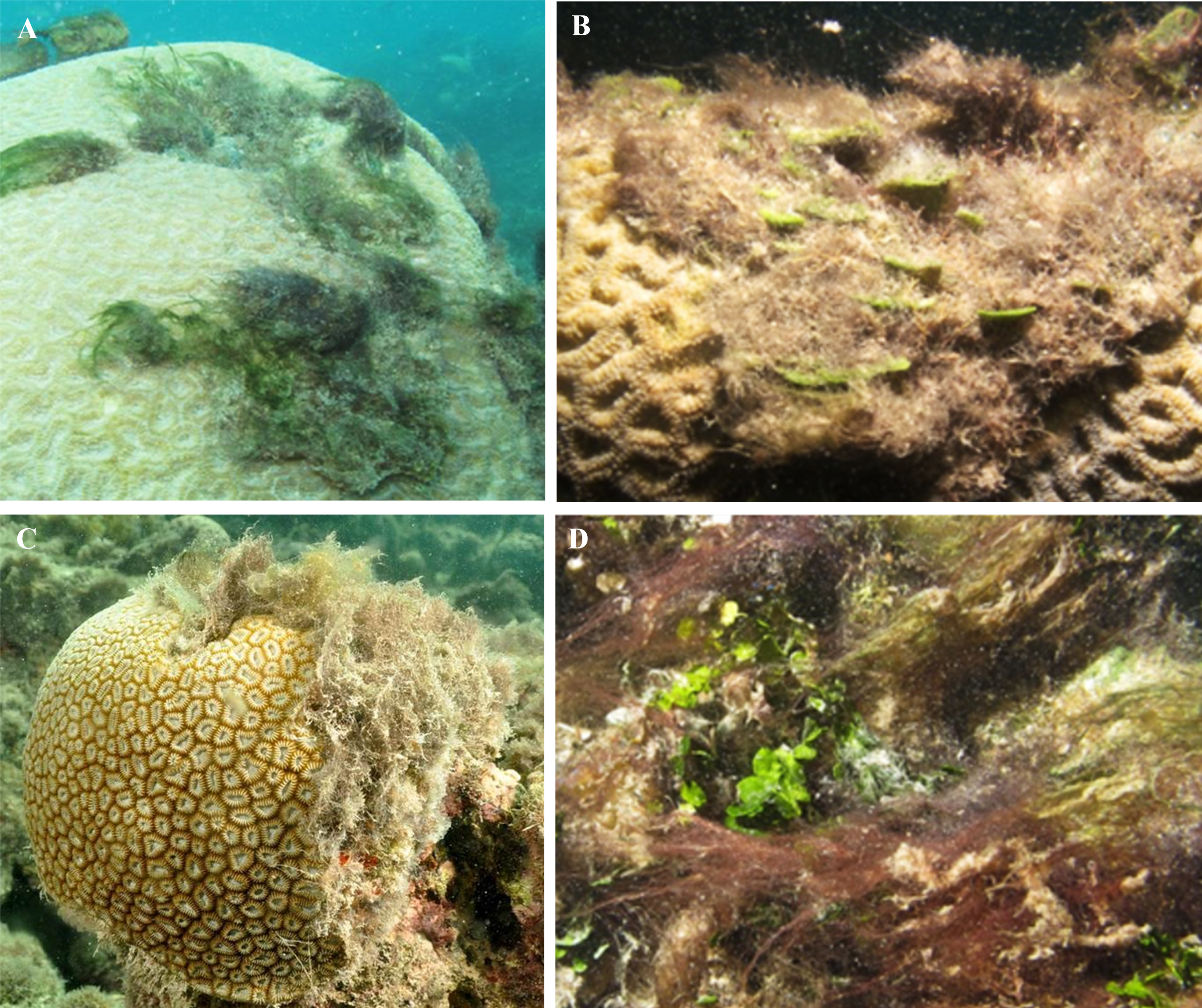

Supplement: S1 Fig — A, B, C, turf growing over Mussismilia corals. D, turf growing over Orbicella coral. (TIF) [file pone.0161168.s001.tif]

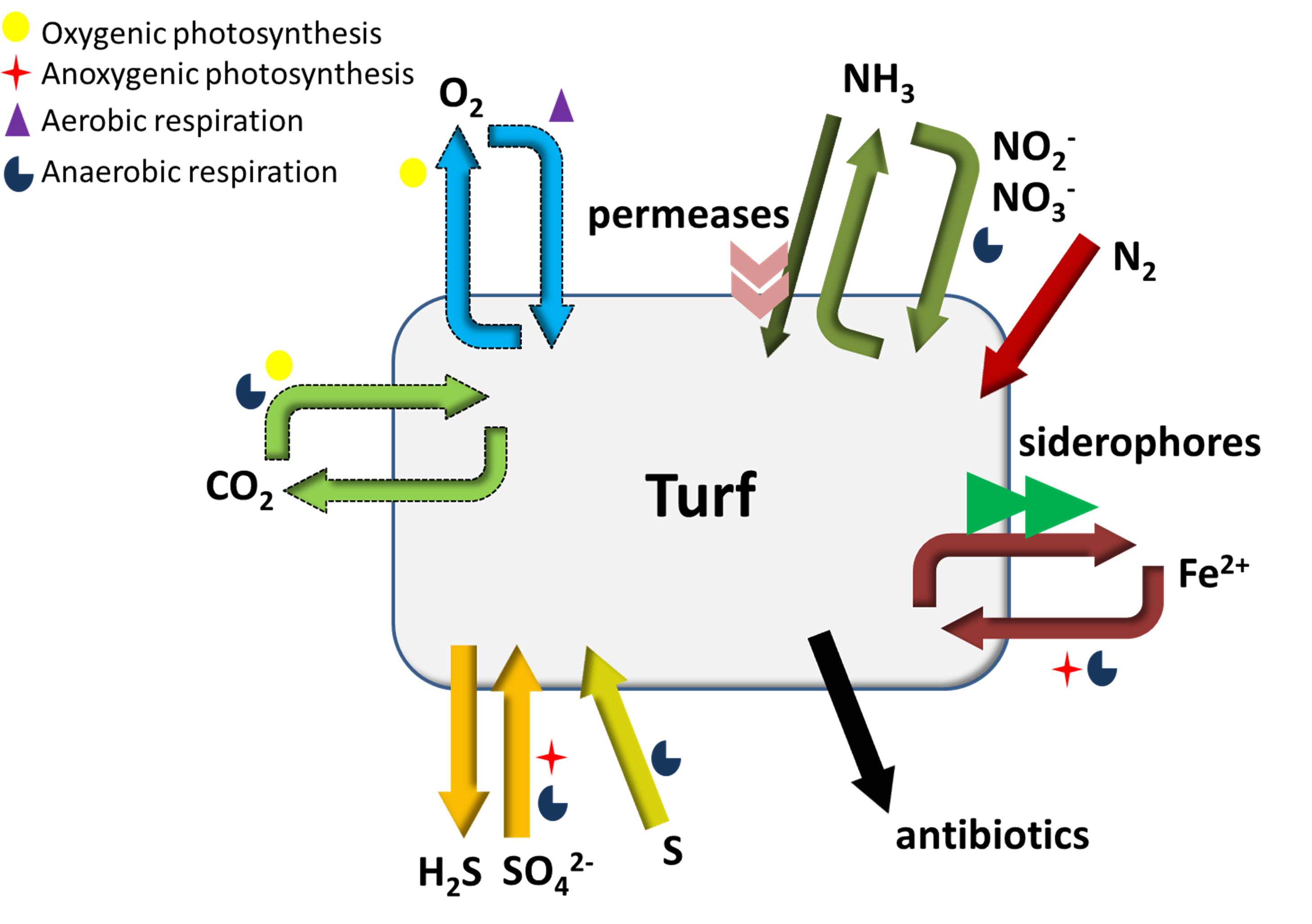

Supplement: S2 Fig — Conceptual model presenting the major metabolisms acting in turf from Abrolhos reefs, which are not a common function in other holobionts (e.g., sulphate reduction and anoxygenic photosynthesis absent in healthy corals). (TIF) [file pone.0161168.s002.tif]
