# Supplementary material for: Taxonomic and Functional Metagenomic Signature of Turfs in the Abrolhos Reef System (Brazil)
Source: PLoS One. 2016 Aug 22;11(8):e0161168. doi: 10.1371/journal.pone.0161168 (PMC4993507; doi:10.1371/journal.pone.0161168)
Supplement: S3 Table — Adonis (PERMANOVA) results of taxonomic composition of turf metagenomes (bacterial order level) based on Bray-Curtis distances with 999 permutations. MS, mean sum of squares; SS, sum of squares. D.f., degrees of freedom; SS, sum of squares; MS, mean sum of squares. (DOCX) [file pone.0161168.s005.docx]

# S3 Table.

|  | Df | SS | MS | pseudoF | R^2^ | *P*-value |
| --- | --- | --- | --- | --- | --- | --- |
| Site | 2 | 0.021 | 0.010 | 0.877 | 0.085 | 0.475 |
| Season | 1 | 0.010 | 0.010 | 0.847 | 0.041 | 0.471 |
| Residuals | 18 | 0.212 | 0.012 |  | 0.874 |  |
| Total | 21 | 0.243 |  |  | 1.000 |  |
