# Supplementary material for: Taxonomic and Functional Metagenomic Signature of Turfs in the Abrolhos Reef System (Brazil)
Source: PLoS One. 2016 Aug 22;11(8):e0161168. doi: 10.1371/journal.pone.0161168 (PMC4993507; doi:10.1371/journal.pone.0161168)
Supplement: S5 Table — Adonis (PERMANOVA) results of functional composition of turf metagenomes (SEED Level 1 Subsystems) based on Bray-Curtis distances with 999 permutations. MS, mean sum of squares; SS, sum of squares. D.f., degrees of freedom; SS, sum of squares; MS, mean sum of squares. (DOCX) [file pone.0161168.s007.docx]

# S5 Table.

|  | Df | SS | MS | pseudoF | R^2^ | *P*-value |
| --- | --- | --- | --- | --- | --- | --- |
| Site | 2 | 0.001 | 0.000 | 1.007 | 0.098 | 0.396 |
| Season | 1 | 0.000 | 0.000 | 0.593 | 0.029 | 0.749 |
| Residuals | 18 | 0.005 | 0.000 |  | 0.873 |  |
| Total | 21 | 0.006 |  |  | 1.000 |  |
