# Supplementary material for: Taxonomic and Functional Metagenomic Signature of Turfs in the Abrolhos Reef System (Brazil)
Source: PLoS One. 2016 Aug 22;11(8):e0161168. doi: 10.1371/journal.pone.0161168 (PMC4993507; doi:10.1371/journal.pone.0161168)
Supplement: S9 Table — Diff., difference in the observed means. (DOCX) [file pone.0161168.s011.docx]

**S9 Table**

|  |  | 95% confidence level interval | |  |
| --- | --- | --- | --- | --- |
|  | Diff. | Lower | Upper | *P* value (adjusted) |
| **Oxygenic** |  |  |  |  |
| rhodolith-coral | 34.074 | 12.172 | 55.975 | 0.001 |
| turf-coral | 18.436 | 1.344 | 35.528 | 0.030 |
| water-coral | 8.235 | -12.042 | 28.512 | 0.696 |
| turf-rhodolith | -15.638 | -34.629 | 3.353 | 0.138 |
| water-rhodolith | -25.838 | -47.740 | -3.937 | 0.015 |
| water-turf | -10.200 | -27.292 | 6.892 | 0.388 |
| **Anoxygenic** |  |  |  |  |
| rhodolith-coral | 0.708 | -1.439 | 2.855 | 0.811 |
| turf-coral | 1.898 | 0.222 | 3.573 | 0.021 |
| water-coral | -0.009 | -1.997 | 1.979 | 1.000 |
| turf-rhodolith | 1.190 | -0.672 | 3.051 | 0.329 |
| water-rhodolith | -0.717 | -2.864 | 1.430 | 0.806 |
| water-turf | -1.907 | -3.582 | -0.231 | 0.020 |
| **Chemolito** |  |  |  |  |
| rhodolith-coral | -2.819 | -24.607 | 18.968 | 0.985 |
| turf-coral | 35.086 | 18.083 | 52.089 | 0.000 |
| water-coral | 12.547 | -7.624 | 32.719 | 0.352 |
| turf-rhodolith | 37.905 | 19.013 | 56.797 | 0.000 |
| water-rhodolith | 15.367 | -6.421 | 37.154 | 0.247 |
| water-turf | -22.538 | -39.541 | -5.536 | 0.005 |
